# Testing a Hypothesis of 12S rRNA Methylation by Putative METTL17 Methyltransferase

**DOI:** 10.32607/actanaturae.25441

**Published:** 2023

**Authors:** A. V. Mashkovskaia, S. S. Mariasina, M. V. Serebryakova, M. P. Rubtsova, O. A. Dontsova, P. V. Sergiev

**Affiliations:** Faculty of Bioengineering and Bioinformatics, Lomonosov Moscow State University, Moscow, 119192 Russian Federation; Institute of functional genomics, Lomonosov Moscow State University, Moscow, 119192 Russian Federation; Department of Chemistry, Lomonosov Moscow State University, Moscow, 119192 Russian Federation; RUDN University, Moscow, 117198 Russian Federation; Belozersky Institute of Physico-Chemical Biology, Lomonosov Moscow State University, Moscow, 119192 Russian Rederation; Center for Molecular and Cellular Biology, Skolkovo Institute of Science and Technology, Moscow, 119192 Russian Federation; Shemyakin-Ovchinnikov Institute of Bioorganic Chemistry, Russian Academy of Sciences, Moscow, 117997 Russian Federation

**Keywords:** mitochondrial ribosome, ribosome assembly factors, methyltransferases, RNA methylation, MALDI-TOF mass spectrometry

## Abstract

Mitochondrial ribosome assembly is a complex multi-step process involving many additional factors. Ribosome formation differs in various groups of organisms. However, there are universal steps of assembly and conservative factors that have been retained in evolutionarily distant taxa. METTL17, the object of the current study, is one of these conservative factors involved in mitochondrial ribosome assembly. It is present in both bacteria and the mitochondria of eukaryotes, in particular mice and humans. In this study, we tested a hypothesis of putative METTL17 methyltransferase activity. MALDI-TOF mass spectrometry was used to evaluate the methylation of a putative METTL17 target – a 12S rRNA region interacting with METTL17 during mitochondrial ribosome assembly. The investigation of METTL17 and other mitochondrial ribosome assembly factors is of both fundamental and practical significance, because defects in mitochondrial ribosome assembly are often associated with human mitochondrial diseases.

## INTRODUCTION

Mitochondrial ribosome assembly involves many factors that act in a strict hierarchy [1, 2]. Disruption of one of the assembly factors can significantly reduce the efficiency of ribosomal particle maturation. One of the conserved mitochondrial ribosome assembly factors, the METTL17 protein of class I SAM-dependent methyltransferases, harbors a mitochondrial localization signal and interacts with the small subunit of the mitochondrial ribosome during assembly [3, 4, 5].

The METTL17 factor plays an important role in mitoribosomal small subunit maturation; during ribosome assembly, METTL17 interacts with several small subunit intermediates at the site where mRNA binding occurs in mature ribosomes [4, 5]. Binding of METTL17 leads to conformational changes in the small subunit’s 12S rRNA region comprising helices 31–34 [4, 5]. In the absence of METTL17, mitochondrial ribosome assembly does not occur in correct fashion. A METTL17 knockout was shown to result in a decrease, not complete cessation, in the methylation level of two nucleotide residues in 12S rRNA [3], which is associated with disruption of the interaction with a mitoribosome assembly intermediate of known RNA methyltransferases [6, 7, 8, 9]. Errors in mitoribosome maturation in the absence of METTL17 lead to defects in mitochondrial translation and mitochondrial respiratory function [3, 4, 5]. At the level of the body, decreased METTL17 synthesis is associated with the development of Friedreich’s ataxia, one of the most common mitochondrial diseases [4].

Obviously, METTL17 is extremely important for correct mitoribosome assembly, but possible methyltransferase activity of this factor has not been studied. The fact is that METTL17 is assigned to the class I SAM-dependent methyltransferase family based on the sequence similarity and the presence of the methyltransferase domain and S-adenosylmethionine binding site in its structure. According to the human and trypanosome METTL17 structures, the METTL17 variants in these species can bind SAM, which is not true for the yeast homolog [4, 5]. These facts suggest that the METTL17 factor has the potential to exhibit methyltransferase activity.

**Fig. 1:**
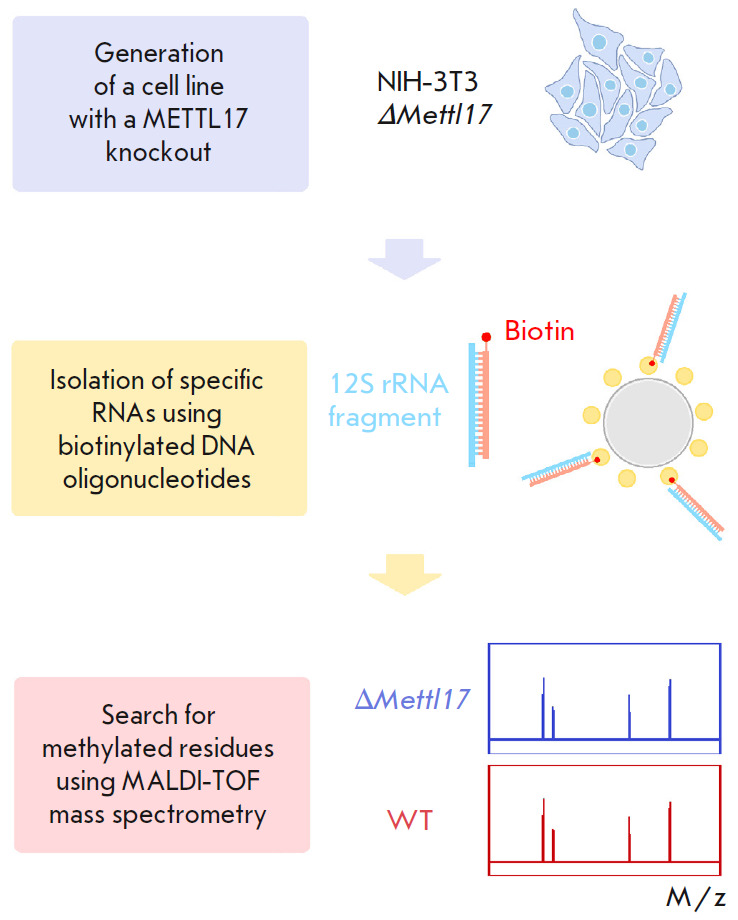
Diagram of the search for the methylation target of the METTL17 mitochondrial factor

We noticed that METTL17 interacts with a 12S rRNA region involving helices 31–34 [4, 5] during assembly and hypothesized that it might modify some nucleotide residue in this rRNA region. There are five known methylation sites in mitochondrial 12S rRNA, each of which is methylated by an appropriate methyltransferase [6, 7, 8, 9, 10, 11, 12]. However, we suspected that some modification might have been overlooked and decided to test a hypothesis that METTL17 methylates a mitochondrial 12S rRNA region comprising helices 31–34. The study flow chart and experiments performed to test the METTL17 target hypothesis are shown in *Fig. 1*.

## EXPERIMENTAL

***Mettl17 *gene inactivation**

The CRISPR-Cas9 system was used to inactivate the* Mettl17 *gene in the NIH-3T3 cell line. The best-ranked guide RNA (5’-GACATTTACCTGTAGAGCCGG-3’) for cleaving the third exon of *Mettl17 *was selected using the Benchling CRISP designing tool (https://benchling. com). The genetic construct was generated using two DNA oligonucleotides containing the guide RNA sequence and adapters for ligation into the plasmid (the guide RNA sequence is shown in gray, the complementary sequence is shown in light gray):

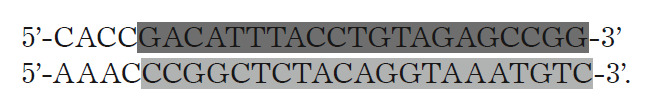



Oligonucleotides were hybridized in T4-DNA ligase buffer (Thermo Scientific, USA): every oligonucleotide was added to a concentration of 1 μM, incubated at 95°C for 5 min, and left to cool to a temperature of 30°C in a closed thermostat. The resulting duplex solution (1 μL) was ligated into the pSpCas9(BB)-2A-GFP plasmid (PX458, Addgene #48138) cleaved at the BpiI restriction endonuclease recognition sites [13]. Competent *Escherichia coli *cells (JM109 strain) were transformed with a ligase mixture, and colonies were grown on a plate supplemented with ampicillin (50 μg/mL). Plasmid DNA was isolated from overnight cultures using a Plasmid Miniprep reagent kit (Evrogen, Russia). The insert in the plasmid was confirmed by Sanger sequencing using a primer for the U6 promoter (5’-GACTATCATATGCTTACCGT-3’).

Wild-type NIH-3T3 cells were transfected with the guide RNA-containing plasmid using the Lipofectamine 3000 reagent (Thermo Scientific). For transfection, 100,000 cells, 500 ng of the plasmid, and 1.5 μL of lipofectamine were used. Twenty-four hours after transfection, cells were selected based on GFP fluorescence using a FACSAria III BD cell sorter; the signal was recorded at absorption/emission wavelengths of 488/530 nm. The selected cells were seeded into a 96-well plate (200 μL of medium per well) for monoclones. Individual monoclones were further cultured in the wells of a 24-well plate. To confirm the* Mettl17 *knockout, total DNA was isolated from the cells using a QuickExtract DNA Extraction Solution (Lucigen). Next, the fragment comprising the cleaved region was amplified by PCR.

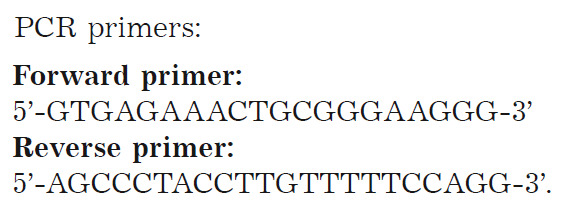



The *Mettl17 *mutation leading to gene inactivation was verified by Sanger sequencing of the amplified fragments.

**Cultivation of cell lines**

Wild-type NIH-3T3 and Δ*Mettl17 *cells were cultured at 37°C and 5% CO_2_ in DMEM/F12 (Gibco) supplemented with FBS to 10% volume and an antibiotic mixture (100 U/mL penicillin and 100 μg/mL streptomycin) in a GlutaMAX (2 mM L-alanine-L-glutamine) solution. The cells were grown in tissue culture flasks (25 cm^2^) for adherent cells. At 90–100% confluency, the cells were subcultured: wild-type cells at a 1:10 dilution and *ΔMettl17 *cells at a 1:4 dilution. The cells were rinsed with PBS and detached with a 1× Trypsin-EDTA solution (Gibco) in PBS. The required number of cells was resuspended in a fresh medium.

For total RNA isolation, large cell volumes were grown in 150 mm Petri dishes. Before cell harvesting, most of the medium was first removed and cells were detached using a culture scraper. The medium with cells was centrifuged at +4°C and 1,000 rpm for 5 min, then the medium was removed, and the cell pellet was frozen and stored at –80°C until analysis.

**Isolation of 12S rRNA fragments and MALDI-TOF mass spectrometry**

Total RNA was isolated using an ExtractRNA reagent (Evrogen). Cell pellets were thawed on ice and homogenized in an ExtractRNA solution (1 mL per 100 mg of cells) in 15-mL tubes (Tissue grinding CKmix50_15ml) using a Precellys Evolution device. Disruption was performed at 6,000 rpm for 20 s; the procedure was run twice, and during the break, the solution was cooled on ice for 5 min. After cell disruption, total RNA was isolated according to the ExtractRNA reagent protocol; the resulting RNA samples were dissolved in miliQ water to a concentration of 5–7 mg/mL.

12S rRNA fragments were isolated using three biotinylated DNA oligonucleotides complementary to the 12S rRNA regions:

**1. **5’-[biotin]GGTTTGCTGAAGATGGCGGTATATAGGCTGAATTAGCAAG-3’

**2. **5’-[biotin]CCCATTTCATTGGCTACACCTTGACCTAACGTTTTTATGT-3’

**3. **5’-[biotin]GCAAGAGATGGTGAGGTAGAGCGGGGTTTATCGATTATAGAACA-3’.

A solution of total RNA (2 mL, 2 mg/mL) and an oligonucleotide (100 pmol/mL) in 6× SSC buffer was incubated in a thermostat at 95°C for 5 min and then cooled in a closed thermostat to 40°C. After hybridization, the solution was treated with RNase T1 (Thermo Scientific) at a concentration of 1 U/mL at 37°C for 1.5 h. After incubation, DNA/RNA duplexes were isolated using Dynabeads M-280 Streptavidin beads (Thermo Scientific), 100 μL of magnetic beads per sample. The magnetic beads were washed three times with 6× SCC buffer. Then, they were added to the solution and incubated at room temperature under stirring for 30 min. After incubation, the magnetic beads were washed successively with 3× SCC buffer (4 times), 1× buffer (3 times), and 0.1× buffer (3 times). Before the last wash, the beads were transferred to a clean tube. RNA was eluted using two techniques: elution with 100 μL of 0.1× SCC buffer containing 6 M urea (70°C, shaking at 1,000 rpm for 5 min) and elution with DNase I (100 μL of DNase solution in 1× DNase buffer, incubation at 37°C in a thermostat under regular stirring for 30 min). The eluate was collected on a magnetic stand and transferred to a clean tube. Then, isopropanol was added to 50%, NH4OAc to 1 M, and 0.5 μL of Glycoblue and left overnight at –20°C.

The next day, RNA was precipitated by centrifugation at the maximum speed (+4°C for 15 min). The pellet was washed with cold 80% ethanol and dried in a thermostat at 42°C. The RNA pellet was dissolved in 1× RNA Loading Dye (Thermo Scientific) and loaded onto a 12% polyacrylamide gel containing 7 M urea. The gel was stained with an ethidium bromide solution. Bands of RNA fragments were cut from the gel, chopped, washed twice with a solution of 25 mM ammonium citrate and 50% acetonitrile, and then dried in 100% acetonitrile. For MALDI-TOF mass spectrometry, the gel chops were air-dried and treated with an RNase T1 solution in 50 mM ammonium citrate at 37°C for 3 h. A 2,5-dihydroxybenzoic acid solution (50 mg/mL) containing 0.5% TFA and 30% acetonitrile was used as a matrix for MALDI mass spectrometry. An amount of 1.5 μL of the matrix was added to 0.5 μL of a citrate solution containing RNA oligonucleotides, and the mixture was applied to the target and dried. The analysis was performed on an Ultraflex III BRUKER instrument equipped with a UV laser (Nd, 335 nm) using positive ion detection.

**Software**

The Mongo Oligo Mass Calculator freeware [14] was used to generate a mass-ordered list of all oligonucleotides produced by RNase T1 digestion of the mitochondrial 12S rRNA regions. This list was calculated using the sequence from the NCBI Public Sequence Bank (https://www.ncbi.nlm.nih.gov/). The house mouse (*Mus musculus*) mitochondrial 12S rRNA sequence was derived from the mitochondrial genome reference sequence (NC_005089).

## RESULTS AND DISCUSSION

In this study, we tested the hypothesis holding that the METTL17 factor methylates a 12S rRNA region with which it interacts during mitochondrial ribosome assembly. First, we generated a cell line with a* METTL17 *knockout to compare the methylation of the 12S rRNA region under normal and METTL17 depletion conditions. A technique of specific RNA isolation with biotinylated DNA oligonucleotides was used to confirm 12S rRNA methylation. RNA fragments were isolated from wild-type and *METTL17 *knockout cells. The isolated and purified RNA samples were analyzed using MALDI-TOF mass spectrometry. Comparison of RNA masses from wild-type and knockout cells with pre-calculated theoretical masses allowed us to test the hypothesis of rRNA methylation by the METTL17 factor.

**Generation of the NIH-3T3 *ΔMettl17 *cell line**

In this study, we used the NIH-3T3 cell line, which is a line of adherent fibroblast-like cells obtained from mouse embryonic tissue. The *Mettl17 *gene was inactivated using a derivative of the plasmid pX458 [13], which encodes components of the CRISPR/Cas9 system (Cas9 protein gene and guide RNA sequence). The guide RNA was selected in such a way as to make a cut at the beginning of exon 3 of the *Mettl17* gene.

**Fig. 2:**
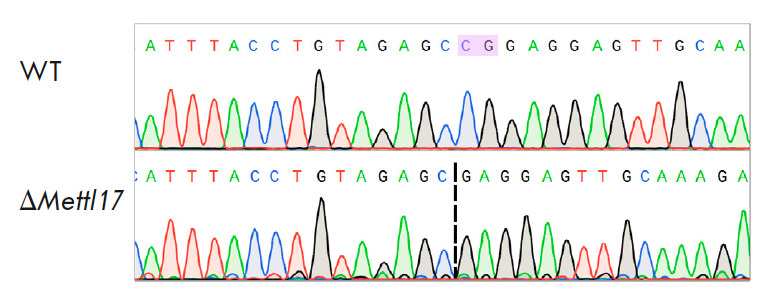
Comparison of the METTL17 protein gene sequence in wild-type (WT) NIH-3T3 and knockout (Δ*Mettl17*) lines, Sanger sequencing

A cell line with a 2 bp deletion in exon 3 of the* Mettl17 *gene was produced. This deletion resulted in a frameshift and the inactivation of the gene. A mutation in the gene was verified using Sanger sequencing (*Fig. 2*).

**Methylation analysis of 12S rRNA fragments**

**Fig. 3:**
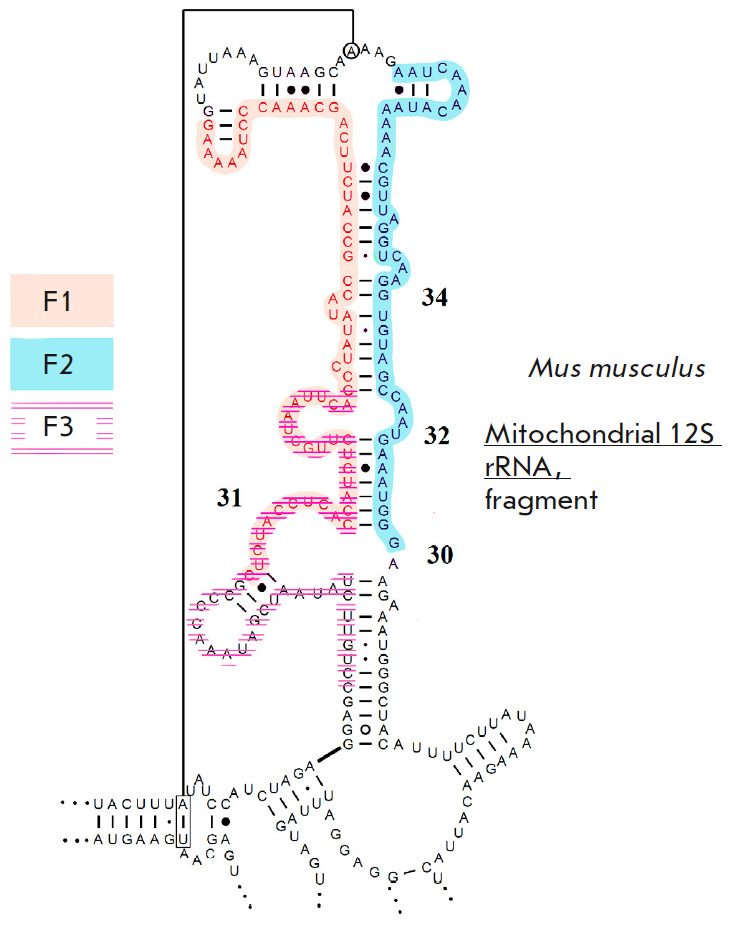
Mouse 12S rRNA fragments analyzed using mass spectrometry (F1, F2, F3). Two fragments (F1, F3) partially overlap. Image adapted from [15]

To confirm methylation, we selected a 12S rRNA region comprising helices 31–34, with which METTL17 interacts. This is a large structured RNA region harboring double-stranded fragments (*Fig. 3*). Owing to this, we decided to divide this region into three fragments (*Fig. 3*). Each fragment was identified and analyzed separately.

**Fig. 4:**
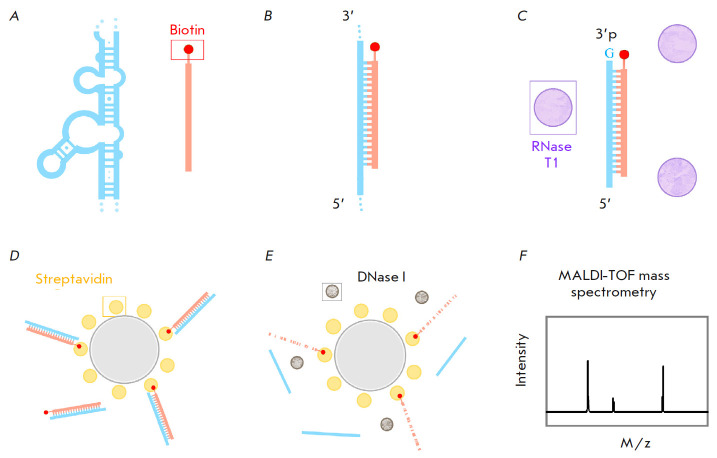
Diagram for isolating RNA fragments using biotinylated oligonucleotides. (*A*) – a 12S rRNA region (blue) and a biotinylated oligonucleotide (coral, biotin – red). (*B*) – after annealing, the secondary structure of rRNA melts, and the rRNA region hybridizes with the oligonucleotide. (*C*) – after treatment with RNase T1, a DNA–RNA duplex remains; RNase T1 cuts ssRNA after guanylic acid residues, leaving a 3’-phosphate. (*D*) – DNA–RNA duplexes bind to magnetic beads through biotin–streptavidin interactions. (*E*) – after treatment with DNase I, DNA is destroyed and RNA occurs in solution. (*F*) – analysis of isolated RNA fragments using MALDI-TOF mass spectrometry

Specific 12S rRNA fragments were isolated using a previously published approach [9, 16, 17], with minor modifications at the elution step. The experiment design is presented in *Fig. 4*. Specific rRNA fragments were isolated using biotinylated DNA oligonucleotides complementary to the 40–50-nt rRNA fragments of interest. After hybridization and the formation of DNA–RNA duplexes, the solution was treated with RNase T1 that excised ssRNA after guanyl residues. Thus, the DNA–RNA duplexes remained in solution, while all ssRNA was destroyed.

**Fig. 5:**
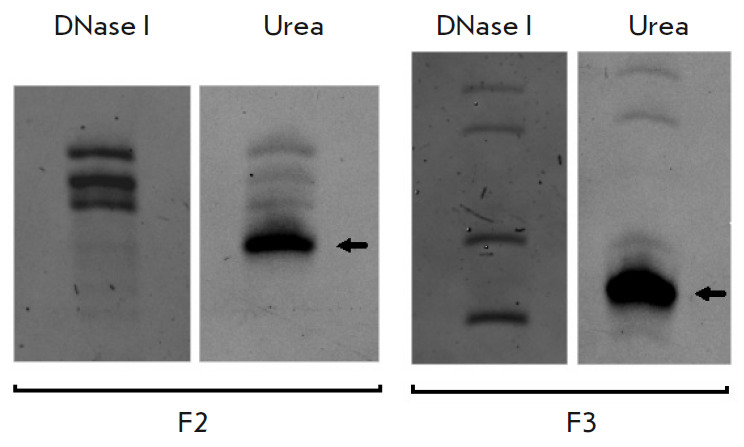
Isolation of RNA fragments (F2, F3) on gel. Elution was performed using urea and DNase I. The DNA oligonucleotide eluted with urea is shown by an arrow

The DNA–RNA duplexes were isolated from the solution using streptavidin magnetic beads. RNA was eluted and separated in polyacrylamide gel to identify fragments of the required length (*Fig. 5*). We tried to elute RNA using a urea solution under heating, in accordance with a previously reported technique [9], as well as using a DNase solution (*Fig. 5*). During elution with urea, not only RNA fragments of interest, but also biotinylated DNA oligonucleotides entered the solution. Treatment with DNase enabled not only eluting RNA but also avoiding DNA in the solution. Loading the eluate on gel provided separation of RNA fragments from DNase molecules: so, the use of enzymatic elution did not complicate the mass spectrometric analysis.

Before the mass spectrometric analysis, bands of RNA fragments were cut from the gel and additionally treated with RNase T1 to digest the RNA into smaller fragments. The maximum fragment weight was 6.2 kDa, and the fragment length was up to 20 nucleotide residues. The theoretical masses of all the fragments were pre-calculated using an online tool [14].

**Fig. 6:**
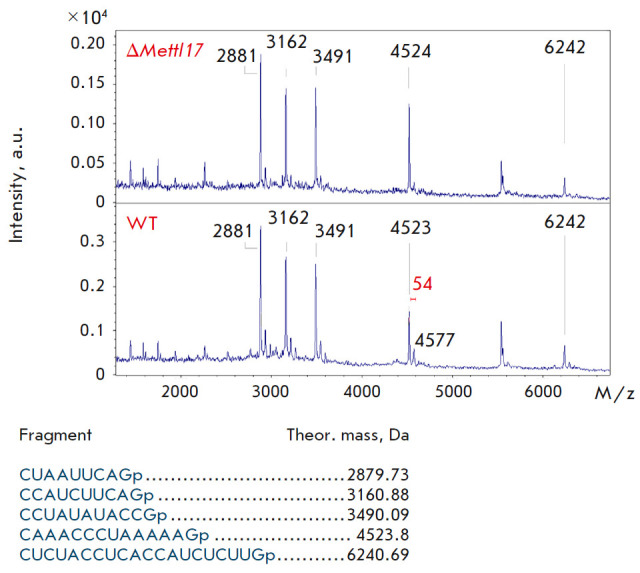
Mass spectra of fragment 1 (peaks of RNA from the *METTL17 *knockout are shown at the top, those from the wild-type line are shown at the bottom). Mass spectra were acquired in linear mode. Theoretically calculated peaks for fragment 1 are labeled

**Fig. 7:**
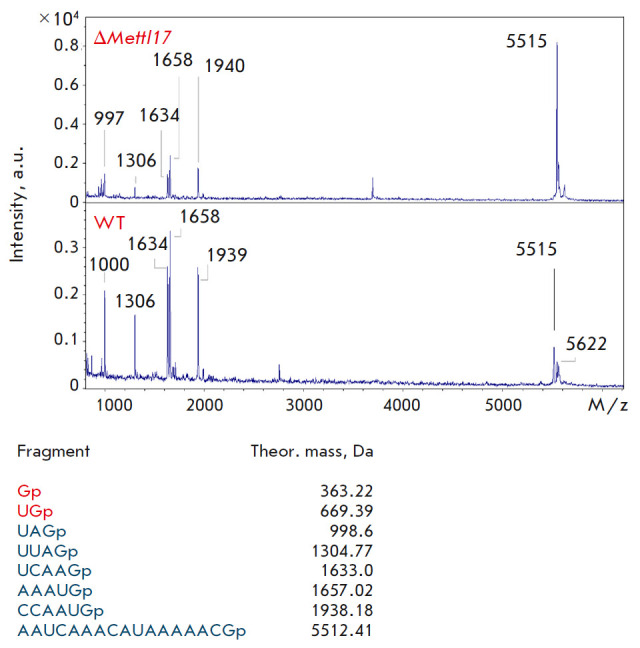
Mass spectra of fragment 2 (peaks of RNA from the *METTL17 *knockout are shown at the top, those from the wild-type line are shown at the bottom). Mass spectra were acquired in linear mode. Theoretically calculated peaks for fragment 2 are labeled

**Fig. 8:**
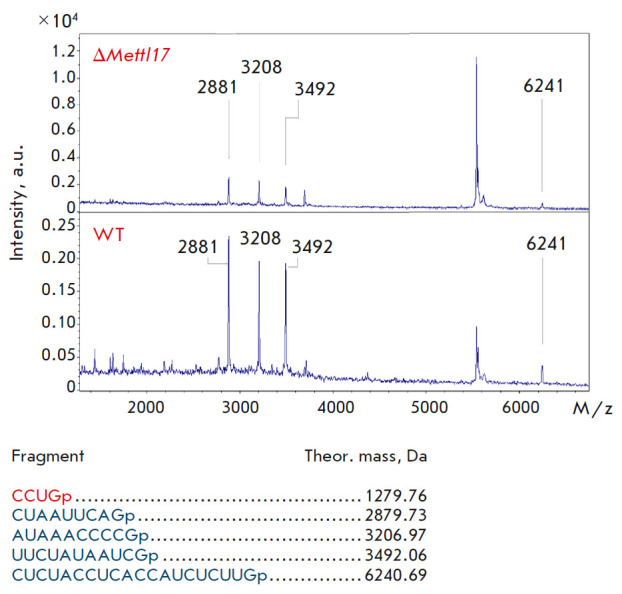
Mass spectra of fragment 3 (peaks of RNA from the *METTL17 *knockout are shown at the top, those from the wild-type line are shown at the bottom). Mass spectra were acquired in linear mode. Theoretically calculated peaks for fragment 3 are labeled

We measured the mass spectra of the hydrolysates of three 12S rRNA fragments from wild-type and Δ*Mettl17 *cells to determine whether an additional methyl group, absent in Δ*Mettl17 *cells, is present in the RNA of wild-type cells. An additional CH_3_ group increases the weight of a fragment by 14 Da. *Figure 6*, *Figure 7*, *Figure 8* show the mass spectra of three fragments; the result for knockout cells is shown on top, and that for wild-type cells is shown at the bottom. Based on the results of the mass spectrometric analysis, we found that the 12S rRNA region interacting with METTL17 was not methylated in wild-type cells, and that fragment masses were not affected by the *METTL17* knockout.

The resulting mass spectra contained all fragments with the predicted masses, with the exception of two short fragments (1–2 nucleotide residues) in the chromatogram of fragment 2 (*Fig. 7*) and a 4-nt fragment in the chromatogram of fragment 3 (*Fig. 8*). In the former case, the fragment mass is too small to be detected. In the latter case, we suggest that the CCUGp fragment was not present in the solution, because it was cleaved off by RNase T1. This region is located at the end of the analyzed fragment and may be cleaved by RNase due to the short length of the double- stranded region. Despite this fact, the entire 12S rRNA region that is in close contact with METTL17 was tested in the experiment. This indicates that METTL17 does not methylate the 12S rRNA region comprising helices 31–34, which leads to a conformational change in this rRNA region.

Therefore, we contend that METTL17 plays a primarily structural role in small mitoribosomal subunit assembly, a role which is not related to methylation. This supports the suggestion [2, 3] that METTL17 is a mitoribosome assembly factor that originates from methyltransferases and retains the characteristic folding and ability to bind SAM at least in some groups of organisms. It probably lost its methyltransferase activity and acts as a structural factor of mitoribosomal small subunit assembly, instead.

## CONCLUSION

In this paper, we tested the hypothesis of methyltransferase activity of the METTL17 protein, a mitochondrial small subunit assembly factor. METTL17 was shown not to methylate the12S rRNA region, with which it comes into contact during assembly, despite the fact that this factor retains the features typical of class I SAM-dependent methyltransferases. We suggest that the METTL17 factor has lost its original function during the evolutionary process and that it instead plays a structural role in mitochondrial ribosome assembly.
